# Supplementary material for: Perceived stress, stigma, and social support among Nepali health care workers during COVID-19 pandemic: A cross-sectional web-based survey
Source: PLOS Glob Public Health. 2022 May 5;2(5):e0000458. doi: 10.1371/journal.pgph.0000458 (PMC10022390; doi:10.1371/journal.pgph.0000458)
Supplement: S1 Table — (DOCX) [file pgph.0000458.s001.docx]

| **S1 Table: Association between stress with independent variables** | | | | |
| --- | --- | --- | --- | --- |
|  |  |  |  |  |
| **Variables** | **Categories** | **Stress** | | **P-value** |
|  |  | **Low** | **High stress** |  |
| **Gender** | Male | 135(61.1) | 86(38.9) | **<0.001** |
|  | Female | 54(34.0) | 105(66.0) |  |
| **Marital status** | Currently Married | 50(37.6) | 83(62.4) | **0.001** |
|  | Currently Unmarried | 139(56.3) | 108(43.7) |  |
| **Age** | Less than 30 years | 36(37.5) | 60(62.5) | **0.008** |
|  | 30-45 years | 135(52.5) | 122(47.5) |  |
|  | 45 years and above | 18(66.7) | 9(33.3) |  |
| **Type of family** | Joint/Extended | 106 (47.5) | 117 (52.5) | 0.31 |
|  | Nuclear | 83 (52.9) | 74 (47.1) |  |
| **Currently working provinces** | Province 1 | 13 (46.4) | 15 (53.6) |  |
|  | Province 2 | 27 (50) | 27 (50) |  |
|  | Bagmati | 47 (48) | 51 (52) |  |
|  | Gandaki | 29 (63) | 17 (37) | 0.54 |
|  | Lumbini | 37 (52.1) | 34 (47.9) |  |
|  | Karnali | 17 (42.5) | 23 (57.5) |  |
|  | Sudhurpaschim | 19 (44.2) | 24 (55.8) |  |
| **Currently staying (in)** | hostel/quarter/rented house | 115 (50.2) | 114 (49.8) |  |
|  | In own home | 74 (49) | 77 (51) |  |
| **Average monthly income** | Less than 40,000 | 104(45.4) | 125(54.6) | **0.03** |
|  | 40,000 and above | 85(56.3) | 66(43.7) |  |
| **Working experience** | Less than 5 years | 55(37.4) | 92(62.6) | **0.001** |
|  | 5 to 10 years | 74(55.6) | 59(44.4) |  |
|  | More than 10 years | 60(60) | 40(40) |  |
| **Stay in isolation** | Yes | 51 (44) | 65 (56) | 0.14 |
|  | No | 138 (52.3) | 126 (47.7) |  |
| **Profession** | Doctor | 59(68.6) | 27(31.4) |  |
|  | Medical Laboratory Personnel | 28(43.1) | 37(56.9) |  |
|  | Nurse | 30(31.9) | 64(68.1) |  |
|  | Paramedic (CMA/HA) | 60(58.8) | 42(41.2) | **<0.001** |
|  | Radiological Professional | 12(36.4) | 21(63.6) |  |
| **Type of hospital currently working** | Medical college/academy | 30 (45.5) | 36 (54.5) |  |
|  | Private Hospital | 43 (47.8) | 47 (52.2) |  |
|  | Public Hospital | 97 (53) | 86 (47) |  |
|  | Other facility with covid19 clinic | 19 (46.3) | 22 (53.7) | 0.66 |
| **Staying away from your family** | Yes | 106 (51.2) | 101 (48.8) | 0.53 |
|  | No | 83 (48) | 90 (52) |  |
| **Training/orientation regarding COVID19** | Yes | 86 (54.8) | 71 (45.2) | 0.48 |
|  | No | 103 (46.2) | 120 (53.8) |  |
