# Supplementary material for: Perceived stress, stigma, and social support among Nepali health care workers during COVID-19 pandemic: A cross-sectional web-based survey
Source: PLOS Glob Public Health. 2022 May 5;2(5):e0000458. doi: 10.1371/journal.pgph.0000458 (PMC10022390; doi:10.1371/journal.pgph.0000458)
Supplement: S2 Table — (DOCX) [file pgph.0000458.s002.docx]

| **S2 Table: Sociodemographic factors associated with perceived stigma** | | | | |
| --- | --- | --- | --- | --- |
|  |  |  |  |  |
| **Variables** | **Categories** | **Stigma** | | **P value** |
|  |  |  |  |  |
|  |  | **No stigma (%)** | **Stigmatized (%)** |  |
| **Gender** | Male | 130 (58.8) | 91 (41.2) | **0.003** |
|  |  |  |  |  |
|  | Female | 117 (73.6) | 42 (26.4) |  |
|  |  |  |  |  |
| **Marital status** | Currently married | 157 (63.6) | 90 (36.4) | 0.42 |
|  |  |  |  |  |
|  | Currently unmarried | 90 (67.7) | 43 (32.3) |  |
|  |  |  |  |  |
| **Age** | Less than 30 years | 60(62.5) | 36(37.5) | 0.33 |
|  |  |  |  |  |
|  | 30-45 years | 166(64.6) | 91(35.4) |  |
|  |  |  |  |  |
|  | 45 years and above | 21(77.8) | 6(22.2) |  |
|  |  |  |  |  |
| **Type of family** | Joint/Extended | 163 (73.1) | 60 (26.9) | **<0.001** |
|  |  |  |  |  |
|  | Nuclear | 84 (53.5) | 73 (46.5) |  |
|  |  |  |  |  |
| **Currently working provinces** | Province 1 | 23 (82.1) | 5 (17.9) | **<0.001** |
|  |  |  |  |  |
|  | Province 2 | 46 (85.2) | 8 (14.8) |  |
|  |  |  |  |  |
|  | Bagmati | 42 (42.9) | 56 (57.1) |  |
|  |  |  |  |  |
|  | Gandaki | 41 (89.1) | 5 (10.9) |  |
|  |  |  |  |  |
|  | Lumbini | 32 (45.1) | 39 (54.9) |  |
|  |  |  |  |  |
|  | Karnali | 29 (72.5) | 11 (27.5) |  |
|  |  |  |  |  |
|  | Sudhurpaschim | 34 (79.1) | 9 (20.9) |  |
|  |  |  |  |  |
| **Currently staying** | In hostel/quarter | 84 (77.1) | 25 (22.9) |  |
|  | In own home/rented home | 163 (60.1) | 108 (39.9) | **0.002** |
| **Average monthly income** | Below 40,000 | 148 (64.6) | 81 (35.4) | 0.85 |
|  |  |  |  |  |
|  | Above 40,000 | 99 (65.6) | 52 (34.4) |  |
|  |  |  |  |  |
| **Working experience** | Less than 5 years | 98 (66.7) | 49 (33.3) | **0.009** |
|  |  |  |  |  |
|  | 5 to 10 years | 96 (72.2) | 37 (27.8) |  |
|  |  |  |  |  |
|  | 10 years and above | 53 (53) | 47 (47) |  |
|  |  |  |  |  |
| **Stay in isolation** | Yes | 76 (65.5) | 40 (34.5) | 0.89 |
|  |  |  |  |  |
|  | No | 171 (64.8) | 93 (35.2) |  |
|  |  |  |  |  |
| **Profession** | Doctor | 60 (69.8) | 26 (30.2) | 0.17 |
|  |  |  |  |  |
|  | Medical Laboratory Personnel | 39 (60) | 26 (40) |  |
|  | Nurse | 67 (71.3) | 27 (28.7) |  |
|  |  |  |  |  |
|  | Paramedic (CMA/HA) | 58 (56.9) | 44 (43.1) |  |
|  |  |  |  |  |
|  | Radiological Professional | 23 (69.7) | 10 (30.3) |  |
| **Type of hospital currently working** | Medical college/academy | 47 (71.2) | 19 (28.8) | **0.003** |
|  | Private Hospital | 71 (78.9) | 19 (21.1) |  |
|  |  |  |  |  |
|  | Public Hospital | 106 (57.9) | 77 (42.1) |  |
|  |  |  |  |  |
|  | Other facility with covid19 clinic | 23 (56.1) | 18 (43.9) |  |
| **Staying away from your family** | Yes | 144 (69.6) | 63 (30.4) | **0.04** |
|  |  |  |  |  |
|  | No | 103 (59.5) | 70 (40.5) |  |
|  |  |  |  |  |
| **Got training/orientation regarding COVID19** | Yes | 90 (57.3) | 67 (42.7) | **0.008** |
|  |  |  |  |  |
|  | No | 157 (70.4) | 66 (29.6) |  |
| **Receive the vaccine for COVID19** | Yes | 173 (65.3) | 92 (34.7) | 0.86 |
|  |  |  |  |  |
|  | No | 74 (64.3) | 41 (35.7) |  |
|  |  |  |  |  |
| **Get infected by the COVID19** | Yes | 38 (57.6) | 28 (42.4) | 0.26 |
|  |  |  |  |  |
|  | No | 209 (66.6) | 105 (33.4) |  |
|  |  |  |  |  |
