# Supplementary material for: Perceived stress, stigma, and social support among Nepali health care workers during COVID-19 pandemic: A cross-sectional web-based survey
Source: PLOS Glob Public Health. 2022 May 5;2(5):e0000458. doi: 10.1371/journal.pgph.0000458 (PMC10022390; doi:10.1371/journal.pgph.0000458)
Supplement: S3 Table — (DOCX) [file pgph.0000458.s003.docx]

| **S3 Table: Association between sociodemographic factors and social support** | | | | |
| --- | --- | --- | --- | --- |
|  |  |  |  |  |
| **Variables** | **Categories** | **Social support** | | **P-value** |
|  |  |  |  |  |
|  |  | **Poor** | **Moderate/strong** |  |
|  |  |  |  |  |
| **Gender** | Male | 89(40.3) | 132 (59.7) | 0.07 |
|  |  |  |  |  |
|  | Female | 79(49.7) | 80 (50.3) |  |
|  |  |  |  |  |
| **Marital status** | Currently unmarried | 63 (47.4) | 70 (52.6) | 0.36 |
|  | Currently married | 105(42.5) | 142 (57.5) |  |
|  |  |  |  |  |
| **Age** | Less than 30 years | 45(46.9) | 51 (53.1) | 0.65 |
|  |  |  |  |  |
|  | 30-45 years | 113(44.0) | 144 (56.0) |  |
|  |  |  |  |  |
|  | 45 years and above | 10(37) | 17 (63) |  |
|  |  |  |  |  |
| **Type of family** | Joint/Extended | 114(51.1) | 109 (48.9) | **0.001** |
|  |  |  |  |  |
|  | Nuclear | 54(34.4) | 103 (65.6) |  |
|  |  |  |  |  |
| **Currently working provinces** | Province 1 | 13(46.4) | 15 (53.6) | 0.99 |
|  |  |  |  |  |
|  | Province 2 | 24(44.4) | 30 (55.6) |  |
|  |  |  |  |  |
|  | Bagmati | 40(40.8) | 58 (59.2) |  |
|  |  |  |  |  |
|  | Gandaki | 21(45.70 | 25 (54.3) |  |
|  |  |  |  |  |
|  | Lumbini | 32(45.1) | 39 (54.5) |  |
|  |  |  |  |  |
|  | Karnali | 19(47.50 | 21 (52.5) |  |
|  |  |  |  |  |
|  | Sudhurpaschim | 19(44.2) | 24 (55.8) |  |
|  |  |  |  |  |
| **Currently staying** | In hostel/rented house/quarter | 116(50.7) | 113 (49.3) | **0.002** |
|  | In own home | 52(34.4) | 99 (65.6) |  |
|  |  |  |  |  |
| **Average monthly income** | Below 40,000 | 119 (52) | 110(48.0) | **<0.0001** |
|  |  |  |  |  |
|  | Above 40,000 | 49 (32.5) | 102(67.5) |  |
|  |  |  |  |  |
| **Working experience** | Less than 5 years | 72 (49.0) | 75 (51) | 0.17 |
|  |  |  |  |  |
|  | 5 to 10 years | 59(44.4) | 74 (55.6) |  |
|  |  |  |  |  |
|  | More than 10 years | 37(37) | 63 (63) |  |
|  |  |  |  |  |
| **Stay in isolation** | Yes | 55 (47.4) | 61 (52.6) | 0.41 |
|  |  |  |  |  |
|  | No | 113 (42.8) | 151 (57.2) |  |
|  |  |  |  |  |
| **Staying away from your family** | Yes | 101 (48.8) | 106 (51.2) | **0.04** |
|  |  |  |  |  |
|  | No | 67 (38.7) | 106 (61.3) |  |
|  |  |  |  |  |
| **Got training/orientation regarding COVID19** | Yes | 48 (30.6) | 109 (69.4) | **<0.001** |
|  |  |  |  |  |
|  | No | 120 (53.8) | 103 (46.2) |  |
| **Receive the vaccine for COVID19** | Yes | 109 (41.1) | 156 (58.9) | 0.07 |
|  |  |  |  |  |
|  | No | 59 (51.3) | 56 (48.7) |  |
|  |  |  |  |  |
| **Health Professional** | Doctor | 18 (20.9) | 68 (79.1) | **<0.001** |
|  |  |  |  |  |
|  | Medical Laboratory | 31 (47.7) | 34 (52.3) |  |
|  |  |  |  |  |
|  | Nurse | 48 (51.1) | 46 (48.9) |  |
|  |  |  |  |  |
|  | Paramedics | 50 (49) | 52 (51) |  |
|  |  |  |  |  |
|  | Radiological | 21 (63.6) | 12 (36.4) |  |
|  |  |  |  |  |
| **Type of hospital currently working** | Public Hospital | 73 (39.9) | 110 (60.1) | 0.36 |
|  |  |  |  |  |
|  | Medical College | 29 (43.9) | 37 (56.1) |  |
|  |  |  |  |  |
|  | Private Hospital | 46 (51.1) | 44 (48.9) |  |
|  |  |  |  |  |
|  | Other facility with covid19 clinic | 20 (48.8) | 21 (51.2) |  |
| **Got infected by COVID19** | Yes | 33 (50) | 33 (50) | 0.39 |
|  |  |  |  |  |
|  | No | 134 (42.7) | 180 (57.3) |  |
|  |  |  |  |  |
