# Supplementary material for: Perceived stress, stigma, and social support among Nepali health care workers during COVID-19 pandemic: A cross-sectional web-based survey
Source: PLOS Glob Public Health. 2022 May 5;2(5):e0000458. doi: 10.1371/journal.pgph.0000458 (PMC10022390; doi:10.1371/journal.pgph.0000458)
Supplement: Attachment — Submitted filename: Rebutal_letter_stress_stigma_rev.docx [file pgph.0000458.s004.docx]

8^th^ March 2022

To

The Editor

PLOS Global Public Health

Re: [PGPH-D-21-00826] “*Perceived stress, stigma and social support among Nepalese health care workers during COVID-19 Pandemic: a cross-sectional web-based survey*”

Dear Dr, Behdin Nowrouzi-Kia,

Thank you so much for your and reviewer’s comments on our manuscript. We have carefully revised the manuscript in response to the extensive and insightful comments we received. We are pleased that there is interest in the topic and are grateful for the constructive comments.

We have addressed the comments point by point and have made corresponding changes in the manuscript in track changes. Appended below, please find the list of all reviewers' comments and our response to each point.

We hope this revised version will be suitable for publication in PLOS Global Public Health.

Yours sincerely,

Kiran Paudel

[Kiranpaudel59@gmail.com](mailto:Kiranpaudel59@gmail.com)

Corresponding author.

**Response to Reviewer’s comments**

**Reviewer#1:**

**Comment#1:** It is well known that HCW are subjected to high stress and stigma as well as with poor social support during the COVID-19 pandemic. There is one study conducted in Nepal itself and published in PLOS ONE. Similar studies were conducted in several other nations as well (to name a few India, China, Jordan, Egypt, Italy, USA etc.). Even there are three review studies published on the same topic. Hence what this study adds to the existing evidence? The introduction lacks justification of conducting this study.

**Reply#1:** Thank you for the suggestion to highlight the importance of this research. We have kindly added the importance of this research on introduction section ***(Page no 3 and 4)*** as:

“Previous studies conducted Nepal did not look into stigma, social support and stress despite the many studies in Nepal included mental health outcome and fears. There is scant information about stigma and social support relating to stress, which could be interesting to look at low-and middle income countries. A timely assessment of the stress, stigma and social support during emergencies will help the management to respond and reduce psychological distress, and help in making public aware about the harmful impact of stigmatization and importance of social support during such humanitarian situation in the future and in such pandemic. “

Khanal, P., Devkota, N., Dahal, M. *et al.* Mental health impacts among health workers during COVID-19 in a low resource setting: a cross-sectional survey from Nepal. *Global Health* **16,** 89 (2020).

Kafle K, Shrestha DB, Baniya A, Lamichhane S, Shahi M, Gurung B, et al. (2021) Psychological distress among health service providers during COVID-19 pandemic in Nepal. PLoS ONE 16(2): e0246784.

Khanal P, Paudel K, Devkota N, Dahal M, Mishra SR, Joshi D (2021) Corona virus fear among health workers during the early phase of pandemic response in Nepal: A web-based cross-sectional study. PLOS Glob Public Health 1(12): e0000083.

**Comment#2**: The sample size computation was based on estimation of a proportion. With an anticipated proportion of 0.56 and absolute precision of 5%, the minimum sample size required for 95 % confidence level is 379. Incorporating 10% non-response rate, the final sample size is 422. However, the authors mentioned the final sample size as 404 which is not correct.

**Reply#2:** Thank you for correcting us. Now, we rewrite in methods section’s sampling ***(Page no.5)*** as:

“The expected proportion of perceived stress of COVID-19 among healthcare providers was taken as 56% from a similar study conducted in Oman (13) and the sample size was calculated using the formula; n = z^2^ pq/d^2^, where p = 0.56, q = 0.44, z = 1.96 at 95% confidence interval, and d = 0.05 is 379. After assuming 10% as the non-response rate, the final calculated sample size was 422.”

**Comment#3:**The mean perceived stress score (PSS) is considered as the cut-off for defining the low and high stress. This is not a good practice to dichotomize a continuous variable based on location parameters like mean or median. There are several articles published that discussed about the danger of use this practice.

**Reply#3:** Thank you for the suggestions. Our analysis is based on similar studies done by Abinet Teshome et al. in Ethiopia published in PLOS One.

We opted for defining outcome variables based on similar studies. Our main purpose is to see factors associated with the presence of stress, stigma, and social support. After your suggestion, we also analyzed data on continuous variables, but no such difference in findings was observed.

Teshome A, Shegaze M, Glagn M, Getie A, Tekabe B, Getahun D, et al. (2021) Perceived stress and associated factors among health care professionals working in the context of COVID-19 pandemic in public health institutions of southern Ethiopia 2020. PLoS ONE 16(6): e0252809. <https://doi.org/10.1371/journal.pone.0252809>

**Comment#4**: The MERS-CoV cut-off used was 26. What was the basis of using this cut-off?
In page no. 6 line no. 114, “After excluding the 24 samples …” the use of “24 samples” is wrong. Instead, it should be “24 participants”.

**Reply#4:** Thank you for the suggestion; we rephrased 24 samples as 24 participants.

Mean a cut-off score of 26 was used to categorize stigmatized as done in a previous study. We have now cited it in the manuscript methods, measures section ***(Page no 6)*** and the text can be now read as below:

“The questionnaire related to stigma among healthcare workers was based on a study used to measure stigma among nursing staff during the Middle East respiratory syndrome coronavirus (MERS-CoV) outbreak (14). The stigma scale comprises 13 items, each of which is scored on a 5-point Likert scale. The total score ranges between 0 and 52, with a higher score indicating that the HCWs perceived greater stigma. For further analysis a mean cut-off score of 26 was used to categorize stigmatized (15).”

**Comment#5**: those variables which were significant in univariate analysis were only included in multiple logistic regression. This is not a good practice as there may be some non-significant variables with P value close to 0.05 which may be clinically important or may become significant in multiple logistic regression. The authors should have used P<0.2 or P<0.25 as a cut-off for inclusion in multivariable analysis instead of P<0.05 obtained in univariate analyses.

**Reply#5:** Thank you for the suggestion. We have done accordingly.

As many traditional levels such as p value of less than 0.05 fail to identify variable known to be important, we put variables, which were significant at 0.1. Brusac Z et al have suggested similar strategy in their article. Link to the article is: <https://doi.org/10.1186/1751-0473-3-17>

Any variables that did not show any association at the 10% significance level were not included further in the model. After doing so, the result on social support changed, and we changed the table in the manuscript ***(Page no 14, 15***). However, the results on stress and stigma did not change.

**Comment#6**: Use of the term “multivariate” is wrong instead it should be “multivariable”.

**#Reply#6:** Thank you for the suggestion; we rephrased as multivariate to multivariable ***(Page no 7).***

**Comment#7:** In Table 3 the perceived stress was categorized in to low, moderate and high. But only one cut-off (based on the mean score of 26) was used. So how three categories were formed?
There were 191 participants with high perceived stress (based on Table 4) which constitute 50.3% of the total sample size. Since this is a cross sectional study, the appropriate summary measure is prevalence ratio (PR) instead of odds ratio (OR). When the event rate is high (more than 10%), the OR obtained using logistic regression over estimates the PR. In such cases modified Poisson regression with robust variance estimation is the suggested analysis which gives the PR and corresponding 95% CI’s.

**Reply#7:** Thank you for the concern. There were some studies, which have categorized in two as stress and no stress taking mean by taking references of previous studies like a study of Ali O Malik and et al. (<https://jamanetwork.com/journals/jamanetworkopen/fullarticle/2767517>). A similar study like ours by Pratik Khanal and et al. among health care workers during the COVID-19 pandemic also analyzed similarly categorizing firstly into three and for multivariable making category two. (<https://globalizationandhealth.biomedcentral.com/articles/10.1186/s12992-020-00621-z> ).

Also the articles published in General Psychiatry by Nisha Kader and et al (<https://annals-general-psychiatry.biomedcentral.com/articles/10.1186/s12991-021-00363-1> ) assessing the perceived stress also categorized stress like ours as 0 to 13, 14 to 26 and 27 to 40 considered low, moderate, and high level of perceived stress. Even if event is high (more than 10%), odds ratio was obtained using logistic regression. In addition, the above-mentioned all articles presented the odds ratio in analysis. A study by H. Mohamed Aly and et al. assessing the perceived stress among health care workers during the covid-19 pandemic also classified stress like ours (<https://bmjopen.bmj.com/content/11/4/e045281>).

**Comment#8**

In Table 4 the unadjusted OR for average income<40000 is 1.6 but the adjusted OR is 0.7. This may be because of multicollinearity. It is better to check this.

**Reply#8:** Thank you for your concern. We checked for multicollinearity after this comment, but it did not show so. Nevertheless, in adjustment, the average monthly income was not statistically significant.

**Comment#9**
In Tables 4 & 5, a few OR’s are with very low values like 0.5 or 0.4 but still not significant. This could be mainly due to small sample size in the respective categories. The authors should consider this while interpreting the results as well as in drawing the conclusions.

**Reply#9:** Thank you for the suggestion. This might be one reason for being so; we now address it in our limitation below. ***(Page no 18)***

**Comment#10**
Interpreting OR as risk is wrong as OR compares the odds of an event between two groups and not the risk.

**Reply#10:** Thank you for the suggestion; we corrected this on the manuscript result section ***(Page no 11)***

**Comment#11**
one of the main limitations of this study is that the sample is not a representative sample as the sampling technique used was non-random sampling. This should be stated in the limitations part.
Some of the latest similar studies were not referred in the manuscript.

**Reply#11:** Thank you for the suggestion; this study depicts the predicament of health-care workers during a pandemic, which could be useful in the future. In the pandemic crisis, non-random sampling was used since researchers were unable to travel and access health care workers and health care personnel were extremely busy at the time. We have written in the limitation section of the manuscript ***(Page 18)*** as:

"Because of a non-random sampling technique, the sample is not representative, which may be a drawback of our study; nevertheless, a random sampling technique can be used to do subsequent research. However, it was one of the most practical ways of conducting research and demonstrating the reality at the time.”

**Reviewer#2**

Thank you for a very fascinating paper. I am making the following suggestions in the hopes that it will strengthen your work further

**1. Introduction**

**Comment#1**

can you reconfigure your introduction to comment on the Nepalese ( or general public's) perception of doctors in usual times i.e. are they typically respected, stigmatized based on specific patient populations they work with and if so what are the known effects of these in Nepal i.e. do people avoid certain fields of practice? Are there known mental health issues? Then I would talk about mental health effects of COVID globally and why your study was conceived i.e. how are these different in LMICÉ in a country where stigma may or may not exist according to area of practice pre-pandemic etc...And then why is it important to know your study results

**Reply#1:** Thank you for the suggestion. Now we considered your comments in the introduction section ***(Page 3, 4).***

“Previous studies conducted Nepal did not look into stigma, social support and stress despite the many studies in Nepal included mental health outcome and fears. There is scant information about stigma and social support relating to stress, which could be interesting to look at in low-income countries. A timely assessment of the stress, stigma, and social support during emergencies will help the management respond and reduce psychological distress and help make the public aware of the harmful impact of stigmatization and the importance of social support during such humanitarian situations in the future this pandemic.“

**2. Methods**

**Comment#2**
Why did you use Oman? Please add detail here. Please add detail of whether you asked about any perceived stigma before the pandemic? Did you resend the survey to get responses? What happened with incomplete surveys i.e. missing data? How were surveys anonymized?

**Reply#2:** Oman was used during the sample calculation because no study has been done so far while planning to carry out this study. We have not added the perceived stigma before the stigma, this could be the limitation of our study, and we included this in limitation.

Incomplete surveys and missing data were excluded from the study that we already mentioned in the methods section ***(Page 7)***.

**Comment#3**

Can you provide more detail of how written consent was separated from data collection? In demographics was vaccine considered one or two doses?

**Reply#3:** Thank you for the suggestion. Detail information of written consent during data collection now written in the methodology section ethical consideration ***(Page no as 7, 8)***

“All the respondents were informed about the aims and objectives of the study by including the written consent form in the questionnaire itself. Written digital consent was taken from study participants prior to completing the survey form. Participants gave their consent by ticking the designated box.”

During the time of data collection, there was only one vaccination. Now we rephrased as one dose in the manuscript.

**3. Results**

**Comment#4**

Please give a sense of what income values means in Nepal context i.e. what is considered poverty line and/or what is equivalent in USD

**Reply#4:** Thank you for the suggestion. Income is commonly conceptualized as the social standing or class of an individual or group. It is often measured as a combination of education, income, and occupation. In the context of Nepal, there is no official socio-economic classification done by the government. Income was one of the major concerns regarding mental health distress. The classification here in this study was done based on the average current salary scale received in a month by healthcare workers**.**

Nepalese Rupees 40,000 becomes 326 United States Dollar that we mentioned in the manuscript methods and results section (***Page no. 6, 9, 11, 14***)

**Comment#5**
- Could you please clarify what you mean that the highest levels of stress were perceived among professions (do you mean professionals?) and that doctors experienced the highest stress? From the table it looks like the physician group was least stressed, am I correct? In addition, that nurses were highest?

**Reply#5:** Thank you for correcting us. Yes, it is professionals. We changed this in the manuscript in every table results . Yes, you are correct.

In the context of the health system of Nepal, doctors are the head of any healthcare facility. Being leading position, they have notified all the information regarding logistic supply, training, etc.; in this way, doctors develop coping mechanisms earlier than other HCWs. This may boost doctors' confidence, allowing them to reduce their stress levels.

**Comment#6**
- please explain what nuclear family vs joint or extended family means -- maybe in methods section when describe data collected. I think this is very important re understanding who is family in Nepal and its implications. Can you comment specifically in the text on the issue of gender here please?

**Reply#6:** Thank you for the suggestion we have explained now in the methods section ***(Page no. 6)*** as;

“Joint/Extended, (Family consisting others like grandparents, siblings etc. including parents and children)

Nuclear (Family consisting of parents and their children only)”

Here we took sex instead of gender thank you for the suggestion; we changed in the manuscript.

**4. Discussion**

**Comment#7**

- Can you comment on the role of education re-pandemic and differences if any received by doctors and nurses on stress levels?

**Reply#7**

Undoubtedly, education is one of the most sustained keys to aware community’s better understanding of the disease spreads without using technical words. The importance of education can be illustrated during the Ebola crisis. The village leaders, traditional healers, religious figures can be vital persons to spread public health messages.

**Comment#8**

You mention coping results in lowered stress in physician - what coping are you referring to?

**Reply#8**

Thank you for the suggestion. Coping results mean coping strategies of stress like training, mediation, health system strengthening to make sure that necessary human workforces and accepting the problems with courage and fight against the pandemic. We corrected this in the discussion section of the manuscript ***(Page no 16)****.*

**Comment#9**

Can you comment on how greater exposure to COVID patients decreased stress levels in other studies and whether you feel your data is aligned with this result? This may be why radiologists are overall more stressed?

**Reply#9:** We have discussed about the greater exposure to COVID-19 patients decreased stress level in discussion ***(Page 16)*** as:

“On the other hand, higher stress among radiological personnel might have been attributed to the limited number of radiological personnel who have to do long time duty in closed spaces (rooms) with fear of being infected. Radiological professionals were not in direct contact with covid-19 patients, they might not be prioritized for PPE, other safety precautions, and the required training at the start of the pandemic that might put them under more stress than medical professionals.”

**Comment#10**
Please address the reasons why stigma may be higher in some provinces, is it education related? Based in fear? More cases per capita? Could it be mitigated in future? Because if HCWs were chased from homes and the community this is not perceived stigma its real-- there is a difference here. Did others have to leave their dwellings too? Do you have that data? What or how did the nature of family change perceptions of stigma?

**Reply#10:** We have addressed the reasons of being stigma difference in provinces on discussion ***(Page no 17)*** as;

“Regarding the location of working institutions, HCWs working in the Bagmati province (the capital region where specialist referral hospitals are located) expressed 3.5 times higher odds of being stigmatized than those working in Sudhurpaschim Province. HCWs had been accused of spreading the coronavirus in the locality and threatening them to vacate their room as soon as possible by the house owners, neighbors even by local leaders and police (25). This type of community behavior might have contributed to the perceiving higher stigma among HCWs. In addition, most of the participants were from Bagmati Province in our study, which might have affected the result. Similarly, HCWs from Karnali Province reported 2.9 higher odds of being stigmatized than the HCWs from Sudhurpaschim Province. Lack of proper knowledge and awareness of the COVID-19 pandemic might have increased the risk of being stigmatized as Karnali province is the most remote having fewer health facilities.”

**Comment#11**
I am not sure the perception of stigma is dissipated because of social supports ( which I would think would help with stress but not the perception of stigma?) or whether it’s just not perceived as strongly because people are already in the family relationships expected of them ie married etc. Can you discuss this please? In other words if you are already different because you are single is the increased stigmatization felt more acutely?

**Reply#11** Thank you for raising the critical concern. As per the reviewer's suggestion, we reanalyzed the data and adjusted it accordingly. The obtained results were discussed likewise ***(Page no 18).***

“Social support is crucial to mitigate mental health burdens and maintain well-being in the HCWs. Its importance is invaluable during the public health emergencies like the COVID-19 pandemic. Various studies have supported the fact that higher social support is associated with better mental well-being during the COVID-19 pandemic (26,27). The HCWs in this study reported higher odds of perceiving social support who were staying at their own house. It is anticipated fear of being infected lies to other members when living in rented houses. Thus, it can be concluded that living in own house might have strong social support.”

**Comment#12**

Same issue with hospitals-- feeling stigmatized is how others perceive you-- it’s not a self-perception thing. So confidence should not play any role i.e. public hospitals. The finding that doctors in a fever hospital felt more stigmatized would likely therefore be more due to the public having a sense (real or not) that these doctors were seeing more COVID patients because COVID is known to cause fever. Therefore these doctors posed a higher risk to public. If doctor was working in a public hospital the public doesn't "know" in the same way that they are seeing a lot of COVID patients.

**Reply#12:** Thank you for the suggestion. We have discussed this in discussion ***(Page 17)*** as

“This study illustrated that HCWs from government hospitals had a lower chance of feeling stigmatized than those currently working at the COVID-19 fever clinic. The reason might be explained as government hospitals have already set up a structure with fine management of human resources. They might have a well-adjusted health system, and the support from the government makes them feel confident. On the other hand, the fever clinics are recently set up to tackle the pandemic with temporary staffing. This might increase fear of uncertainty and chances of being more stigmatized. Furthermore, the fever clinic was the primary point of contact for the COVID-19 patients, so the majority of the infected patients visited. Because of their close contact with covid-19 patients and the potential for disease transmission, health staffs working there may have been ostracized even more.”

**Comment#13**
Living in rented quarters would have been done to protect family would it not? So would the different results be explained that the rented quarters were filled with other HCWs or that HCWs were mostly living alone when renting? Please clarify

**Reply#13:** Thank you for your concern. We clarify this as

Living in a rented apartment would safeguard the family from COVID-19 infections. As a result, during pandemics, they resided in rented quarters or single-occupancy rooms to save their families from infections. Even the health facility's quarters may have filled up during that period.

**Comment#14**
Similarly lower supports were provided to people who received more COVID training-- is this because even family members stood back because they equated receiving training with more exposure to COVID patients?

**Reply#14**: Thank you for bringing this to our attention. Due to the cross-sectional nature of this study, we did not consider the influence of COVID-19 training and social support. More research using a longitudinal study design might provide a better picture. Furthermore, health personnel who may be afraid of covid-19 and believe they lack social support may have gone for training because of the stress.

Lower family supports were given to those who had more COVID-19 training. This might be explained by the notion that health workers went to training to improve their knowledge of COVID-19 and help others comprehend it due to a shortage of social support.

**Comment#15**
If there would be any value in providing more robust public and family (of HCWs) education to help HCWs in the future, how and what would need to be explained? The provision of good living quarters would decrease family support and may result in stigmatization of whole neighborhoods so I would clarify this statement.

**Reply#15:** Thank you so much for your comment. We added this in the conclusion ***(Page no 19)*** also.

Yes, “to combat future pandemic learnt from current COVID 19 health information as the tool of education plays a vital role to act. This should be user driven, proper allocation of resources easy to understand and accessible at the point of use. Addition to this, a good understanding of local disease patterns would help to formulate strategies so that the burden to healthcare workers would be less during emergencies.”
