# Supplementary material for: Perceived stress, stigma, and social support among Nepali health care workers during COVID-19 pandemic: A cross-sectional web-based survey
Source: PLOS Glob Public Health. 2022 May 5;2(5):e0000458. doi: 10.1371/journal.pgph.0000458 (PMC10022390; doi:10.1371/journal.pgph.0000458)
Supplement: Attachment — Submitted filename: Reviewer_Lettter_LG_and_etal..docx [file pgph.0000458.s005.docx]

31^st^ March 2022

To

The Editor

PLOS Global Public Health

Re: [PGPH-D-21-00826] “*Perceived stress, stigma and social support among Nepali health care workers during COVID-19 Pandemic: a cross-sectional web-based survey*”

Dear Dr, Behdin Nowrouzi-Kia,

Thank you so much for your and reviewer’s comments on our manuscript. We have carefully revised the manuscript in response to the extensive and insightful comments we received. We are pleased that there is interest in the topic and are grateful for the constructive comments.

We have addressed the comments point by point and have made corresponding changes in the manuscript in track changes. Appended below, please find the list of all reviewers' comments and our response to each point.

We hope this revised version will be suitable for publication in PLOS Global Public Health.

Yours sincerely,

Kiran Paudel

[Kiranpaudel59@gmail.com](mailto:Kiranpaudel59@gmail.com)

Corresponding author.

**Journal requirements:**

**Requirement#1:** Please ensure that you refer to Table 1 in your text as, if accepted, production will need this reference to link the reader to the table.

**Reply#1:** Thank you for the suggestion and we ensured that our table 1 provided reference link to the reader and rewrite as

“Variables and their definition used in the study are tabulated. (Table 1)” ***(Methods; Measures: Page no.6, 7)***

**Requirement#2:** We notice that your supplementary tables are included in the manuscript file. Please remove them and upload them with the file type 'Supporting Information'. Please ensure that all Supporting Information files are included correctly and that each one has a legend listed in the manuscript after the references list.

**Reply#2**: Thank you for the suggestion we have done accordingly. We have removed them from the manuscript and uploaded them with the file type supporting information. We ensured that all supporting information files are included correctly and that each one has a legend listed in the manuscript after the reference list as:

**“**S1: Association between stress with independent variables

S2: Sociodemographic factors associated with perceived stigma

S3: Association between sociodemographic factors and social support**” *(Page no.22)***

**Requirement#3:** Please review your reference list to ensure that it is complete and correct. If you have cited papers that have been retracted, please include the rationale for doing so in the manuscript text or remove these references and replace them with relevant current references. Any changes to the reference list should be mentioned in the rebuttal letter that accompanies your revised manuscript. If you need to cite a retracted article, indicate the article’s retracted status in the References list and include a citation and full reference for the retraction notice.

**Reply#3:** Thank you for the suggestion we have reviewed our references lists and ensure that it is complete and correct.

**Reviewer’s comments**

The submission entitled "Perceived stress, stigma and social support among Nepali health care workers during COVID-19 Pandemic: a cross-sectional web-based survey" is a resubmission to the PLOS Global Public Health with revisions considering the previous round of comments. I have not reviewed the submission before; therefore, I read the authors’ responses to the previous round of comments and then read the revised manuscript. In my opinion, the authors have satisfactorily addressed the comments mentioned by the previous reviewers.

However, from my perspective, the revised manuscript can be further strengthened using the following comments, especially in the enrichment of literature review.

**Comment#1:** In Introduction, the mental health problems of healthcare workers can be elaborated using the findings on high anxiety level and low trust among Iranian healthcare workers (Hasannia et al., 2021), high prevalence rates of anxiety and depression among African countries (Olashore et al., 2021), negative impacts of stigma on mental health (Lu et al., 2021; Patel et al., 2021), and sleep problems for healthcare providers (Alimoradi et al., 2021; Khan et al., 2021)

**Reply#1:** Thank you for the suggestion, now we have written as:

“During the COVID-19 pandemic, sleep problems, anxiety, and depression appeared to be common, and were linked to higher levels of psychological distress. Anxiety symptoms and fear of COVID-19 were the common predictive factors of distress and sleep problems. Due to the lack of medicine and vaccination of COVID-19 in the beginning, people stigmatized front line health care workers which may have resulted in self-stigmatization and mental health problems. During the COVID-19 pandemic, mental health services, early identification, and treatment of such problems among health care personnel would motivate them to operate their work efficiently and safely. Aside from psychological distress, our research will aid in identifying possible indicators of social support and stigma that may have an impact on the physical and mental health of Nepali health care workers.” ***(Introduction; Page no. 3, 4)***

**References**

Hasannia E, Mohammadzadeh F, Tavakolizadeh M, Davoudian N, Bay M. Assessment of the anxiety level and trust in information resources among iranian health-care workers during the pandemic of coronavirus disease 2019. Asian J Soc Health Behav 2021;4:163-8

Olashore AA, Akanni OO, Fela-Thomas AL, Khutsafalo K. The psychological impact of COVID-19 on health-care workers in African Countries: A systematic review. Asian J Soc Health Behav 2021;4:85-97

Lu MY, Ahorsu DK, Kukreti S, et al. The Prevalence of Post-traumatic Stress Disorder Symptoms, Sleep Problems, and Psychological Distress Among COVID-19 Frontline Healthcare Workers in Taiwan. Front Psychiatry. 2021;12:705657. Published 2021 Jul 12. doi:10.3389/fpsyt.2021.705657

Patel BR, Khanpara BG, Mehta PI, Patel KD, Marvania NP. Evaluation of perceived social stigma and burnout, among health-care workers working in covid-19 designated hospital of India: A cross-sectional study. Asian J Soc Health Behav 2021;4:156-62

Alimoradi Z, Broström A, Tsang HWH, et al. Sleep problems during COVID-19 pandemic and its' association to psychological distress: A systematic review and meta-analysis. EClinicalMedicine. 2021;36:100916. doi:10.1016/j.eclinm.2021.100916

Khan HR, Ashraf F, Ullah I, et al. Cross-cultural prevalence of sleep quality and psychological distress in healthcare workers during COVID-19 pandemic. Brain Behav. 2021;11(11):e2383. doi:10.1002/brb3.2383

**Comment#2**: In Introduction, the sentence "HCWs in the pandemic are more vulnerable to emotional distress as they have a greater risk of exposure to the virus, increased workload, fear of infecting their family and friends, lack of experience in managing the disease, and lack of personal protective equipment (PPE)." can be supported by the following references.

Ref:

Chung GK, Strong C, Chan YH, et al. Psychological Distress and Protective Behaviors During the COVID-19 Pandemic Among Different Populations: Hong Kong General Population, Taiwan Healthcare Workers, and Taiwan Outpatients. Front Med (Lausanne). 2022;9:800962. Published 2022 Feb 15. doi:10.3389/fmed.2022.800962

Rajabimajd N, Alimoradi Z, Griffiths MD. Impact of COVID-19-related fear and anxiety on job attributes: A systematic review. Asian J Soc Health Behav 2021;4:51-5

Zaroushani V, Khajehnasiri F. Errors and preventive measures of health-care systems during COVID-19 pandemic. Asian J Soc Health Behav 2022;5:49-50

**Reply#2:** Thank you for providing the newly published supporting references to this statement. Now we have referenced this in our introduction as;

“HCWs in the pandemic are more vulnerable to emotional distress as they have a greater risk of exposure to the virus, increased workload, fear of infecting their family and friends, lack of experience in managing the disease, and lack of personal protective equipment (PPE) [7-9].” ***(Introduction; Page no.3)***

**References:**

7. Chung GK, Strong C, Chan YH, et al. Psychological Distress and Protective Behaviors During the COVID-19 Pandemic Among Different Populations: Hong Kong General Population, Taiwan Healthcare Workers, and Taiwan Outpatients. Front Med (Lausanne). 2022;9:800962. Published 2022 Feb 15. doi:10.3389/fmed.2022.800962

8. Rajabimajd N, Alimoradi Z, Griffiths MD. Impact of COVID-19-related fear and anxiety on job attributes: A systematic review. Asian J Soc Health Behav 2021;4:51-5

9. Zaroushani V, Khajehnasiri F. Errors and preventive measures of health-care systems during COVID-19 pandemic. Asian J Soc Health Behav 2022;5:49-50

**Comment#3**: In the statement, “To limit non-health worker’s responses to the online survey, forms were only posted in the relevant official social media groups of health workers.” please provide some examples of the official social media groups.

**Reply#3:** Thank you for suggesting us to mention about the official social media groups we rewrite as:

“To limit non-health worker’s responses to the online survey, forms were only posted in the relevant official social media groups of health workers such doctor society of Nepal, registered nurses Nepal, Government doctors association of Nepal, Nepali doctors lounge, Nepali health professional, etc.” ***(Methods; Data collection: Page no.5)***

**Comment#4:** Please mention if the used measures have Nepali version with psychometric properties established.

**Reply#4:** Being our study participant’s health workers who could understand English language we used the questionnaire in the English version being language not barrier. However, for other participants who are not familiar with English language, questionnaire need to translate. Nevertheless, thank you for flagging this issue now we have added this on our limitation as:

“Contextualization of questionnaire items and validation of Nepali version of stigma and social support seems necessary if used to assess stigma and social support in Nepali population in the future.” ***(Limitation; Page no.18)***
